# Supplementary material for: Directed Evolution of Acoustic Reporter Genes Using High-Throughput Acoustic Screening
Source: ACS Synth Biol. 2024 Jul 9;13(7):2215–26. doi: 10.1021/acssynbio.4c00283 (PMC11264329; doi:10.1021/acssynbio.4c00283)
Supplement: Supplementary file 3 — sb4c00283_si_003.pdf [file sb4c00283_si_003.pdf]

Directed Evolution of Acoustic Reporter Genes Using High-Throughput Acoustic Screening

Authors:
Robert C. Hurt[1#], Zhiyang Jin[2#], Mohamed Soufi[3], Katie K. Wong[3], Daniel P. Sawyer[1], Hao K. Shen[3], Przemysław Dutka,[1,3] Ramya Deshpande[3], Ruby Zhang[3], David R. Mittelstein[2], Mikhail G. Shapiro[2,3,4,*]

Affiliations:
[1]Division of Biology and Biological Engineering
[2]Division of Engineering and Applied Science
[3]Division of Chemistry and Chemical Engineering
[4]Howard Hughes Medical Institute
[#]Equal contribution
California Institute of Technology
Pasadena, CA, USA 91125

*Correspondence should be addressed to MGS:

Email: mikhail@caltech.edu
Phone: 626-395-8588

Address: 1200 E. California Blvd, MC 210-41, Pasadena, CA 91125

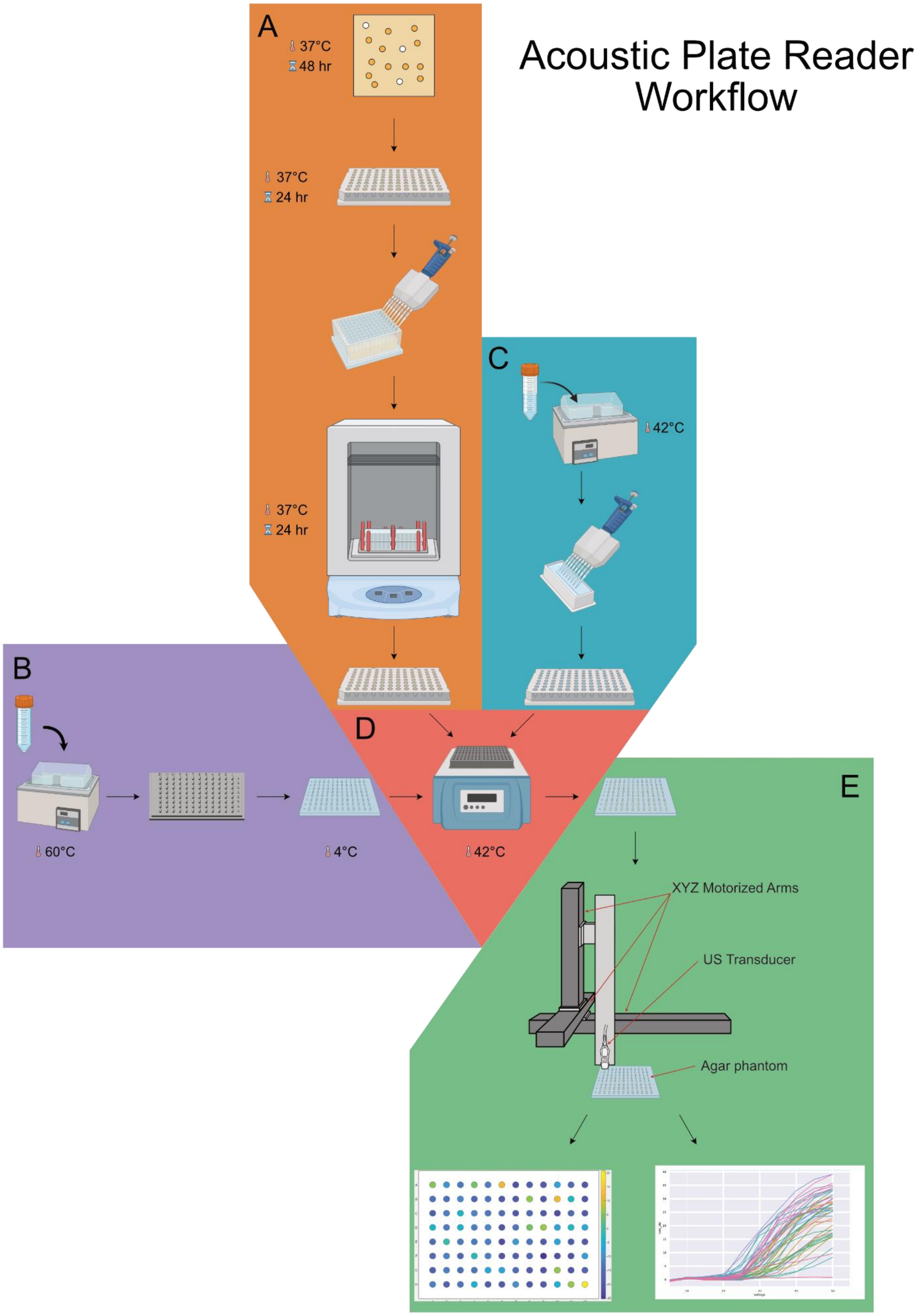

Figure S1. Detailed diagram of the Acoustic Plate Reader workflow. (A) GVs are expressed in *E. coli* as colonies on Petri dishes for 48 hr at 37°C, then colonies are picked into LB and grown to saturation in liquid culture for 24 hr at 37°C. These saturated liquid cultures are then diluted 1:100 into autoinduction LB and expressed for 24 hr at 37°C in 500 uL cultures in deep-well 96-

well plates (square wells are used for maximum culture aeration; USA Scientific 1896-2800). Aliquots of these cultures are aliquoted into an un-skirted 96-well PCR plate for subsequent loading into phantoms. (B) A solution of 2% Ultrapure Agarose (Invitrogen, 16500500) is prepared in 1X PBS and incubated at 60°C for at least 12 hr to degas. Agarose phantoms are then made by pouring 75 mL of this solution into a 96-well phantom mold and incubating at 4°C for 10 min. (C) A solution of 1% low-melting-temperature agarose (Goldbio, A-204-100) is prepared in 1X PBS and incubated at 60°C for at least 12 hr to degas. This solution is then aliquoted into an un-skirted 96-well PCR plate to be used for phantom loading. (D) Phantoms from B are loaded by placing the 96-well PCR plates from A and C into 96-well heat blocks at 42°C, and combining equal volumes of culture and agarose before pipetting into the empty phantom. (E) Phantoms from D are scanned using the acoustic plate reader, which generates US data for each sample and can image up to 12 96-well phantoms in a single scan.

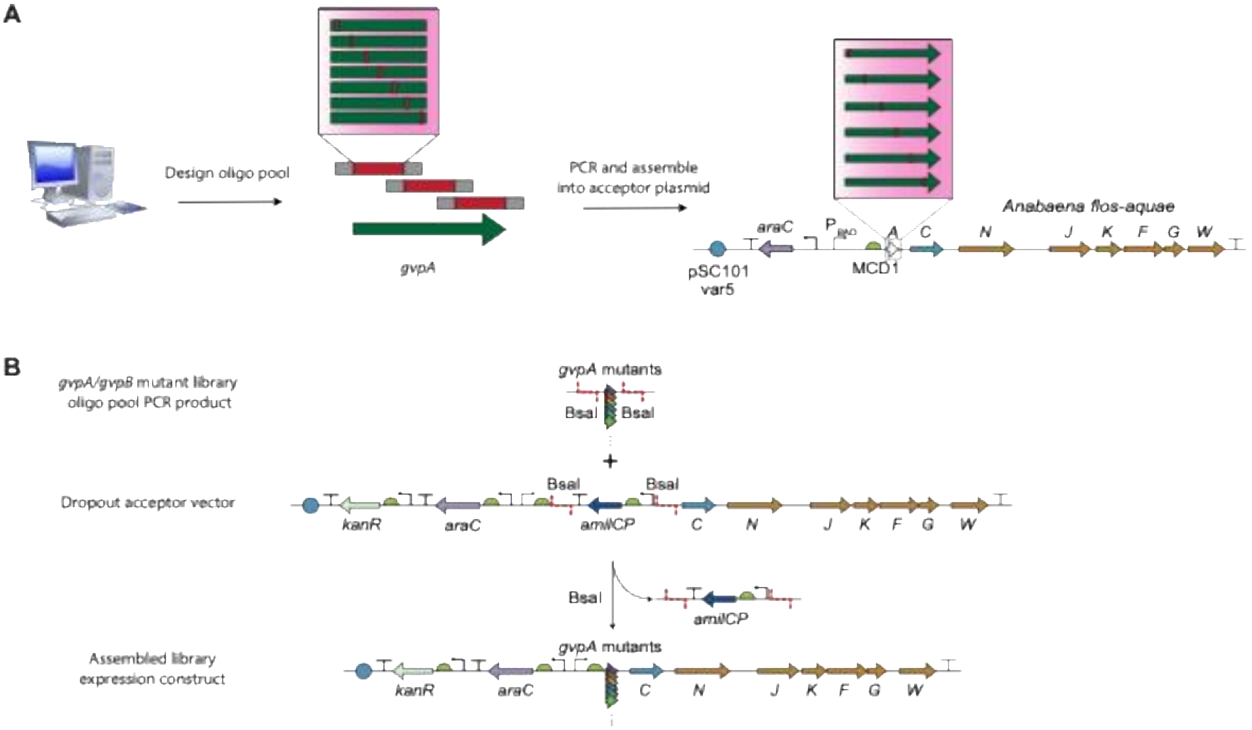

Figure S2. Details of *gvpA*/*gvpB* mutant library construction. (A) Overview of workflow for creating either scanning site saturation or recombination libraries. (B) Details of library assembly via a Golden Gate-based version of cassette mutagenesis (see Methods).

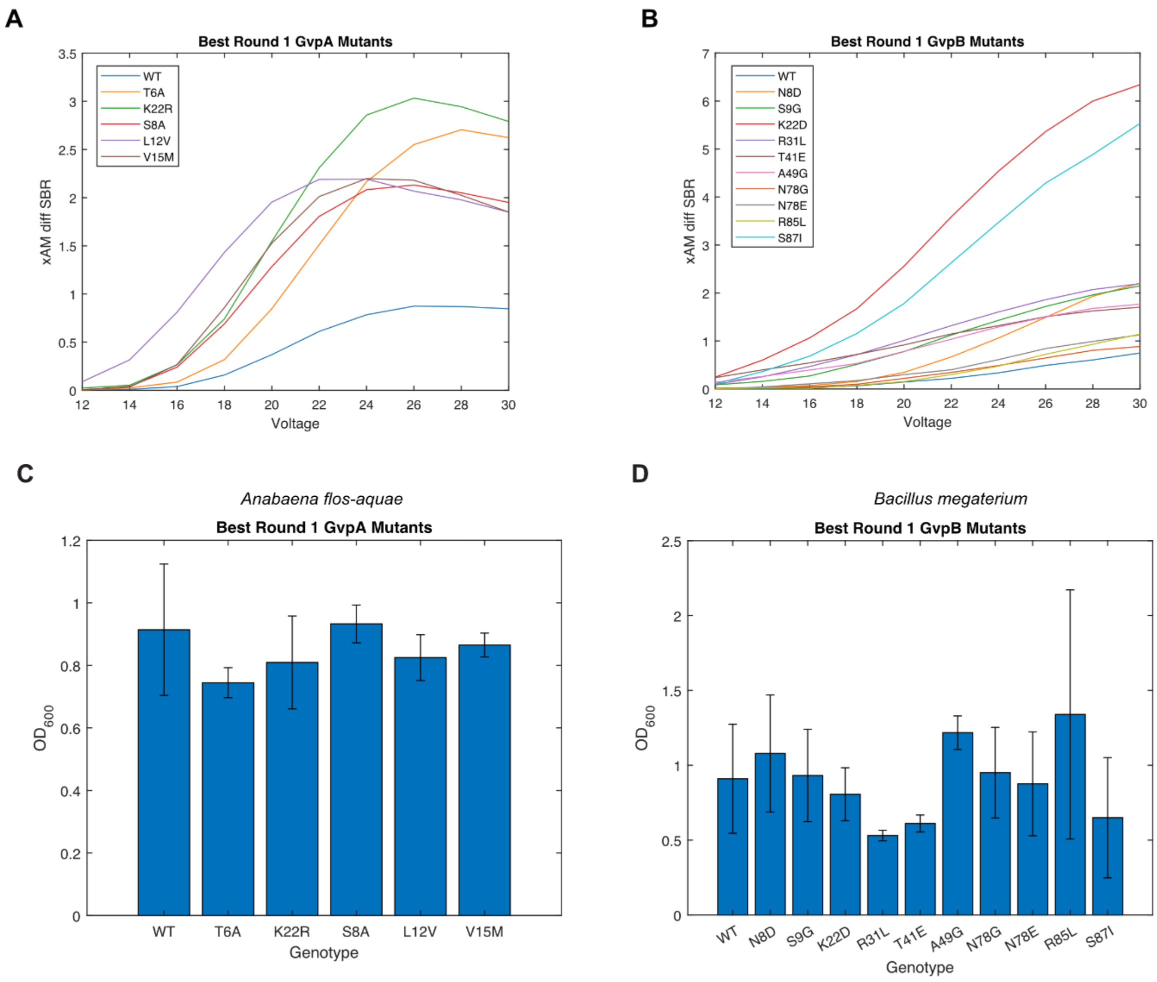

Figure S3. Characterization of the top mutants from Round 1 of evolution. (A-B) xAM difference SBR as a function of pressure for each of the top mutants. N=4 biological samples (each an average of 3 technical replicates). (C-D) OD600 measurements for the mutants shown in A-B. N=4 biological samples.

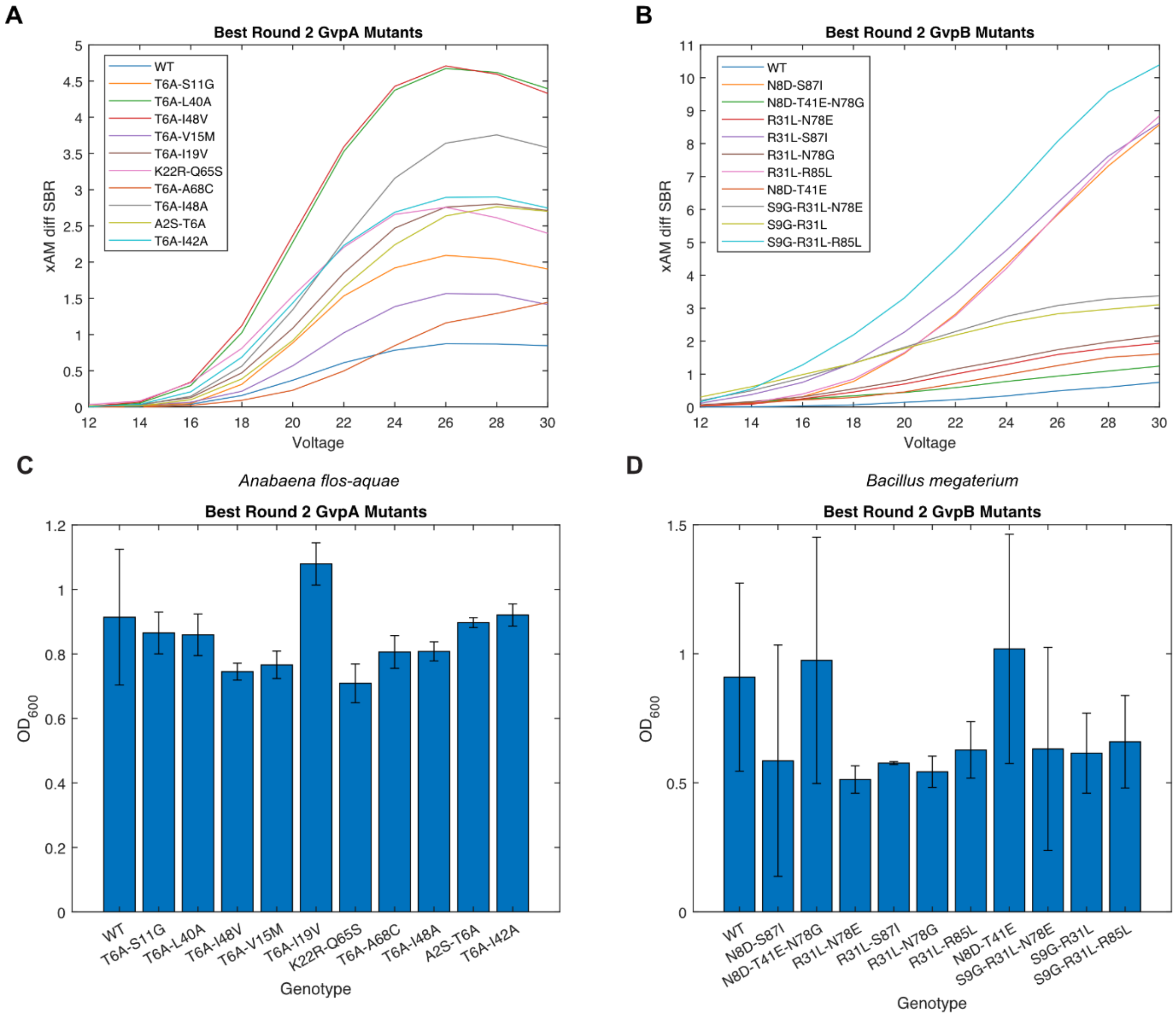

Figure S4. Characterization of the top mutants from Round 2 of evolution. (A-B) xAM difference SBR as a function of pressure for each of the top mutants. N=4 biological samples (each an average of 3 technical replicates). (C-D) OD600 measurements for the mutants shown in A-B. N=4 biological samples.

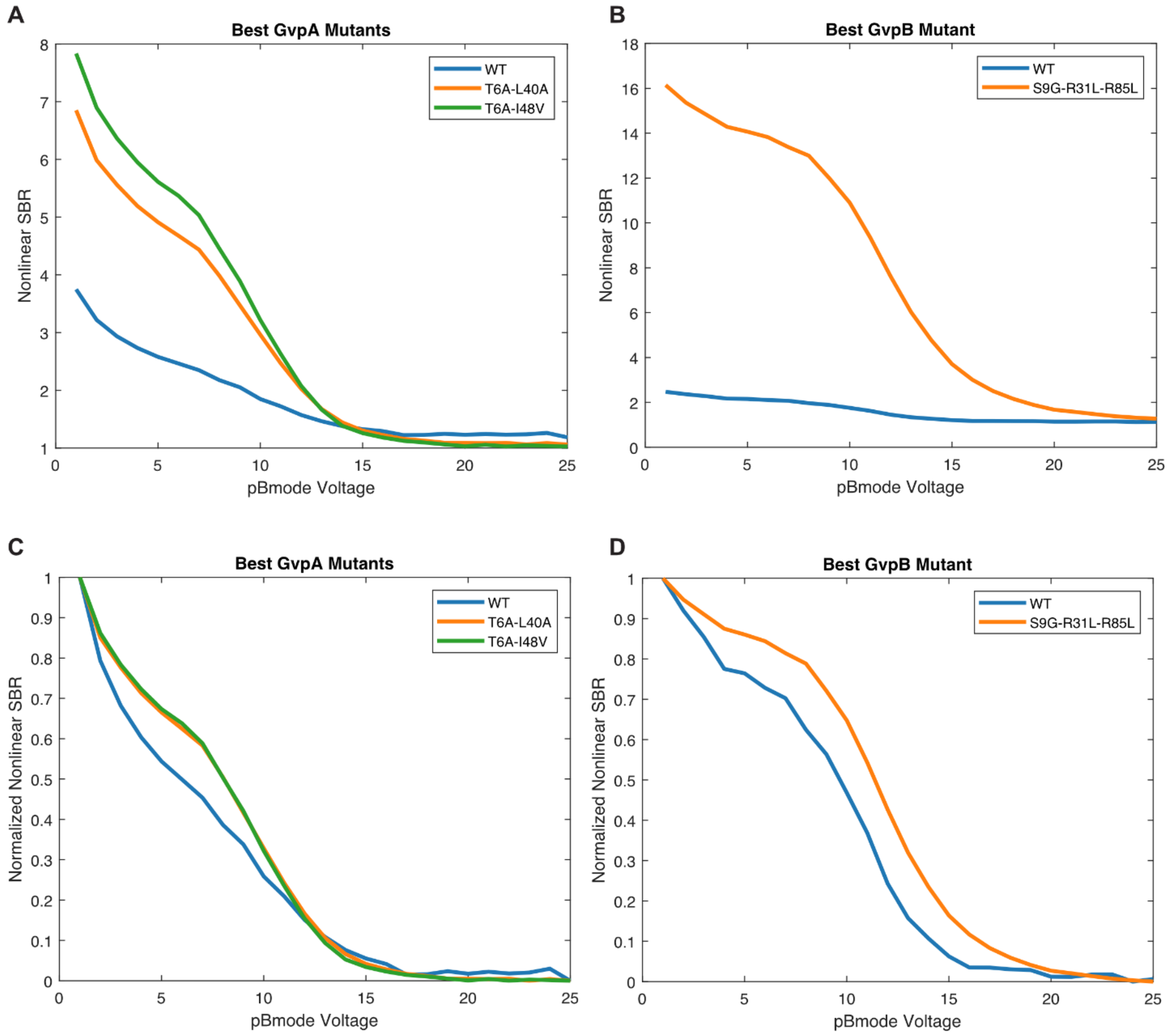

Figure S5. Acoustic collapse pressure curves for the best mutants identified in this study. Acoustic pressure was applied using a parabolic Bmode pulse sequence with increasing voltages, and nonlinear signals were acquired using xAM at 3.1 MPa after each voltage step. (A-B) xAM acoustic collapse pressure curves for the top-performing mutants identified in this study. (C-D) Data from A-B normalized to the same min and max. N=4 biological samples.

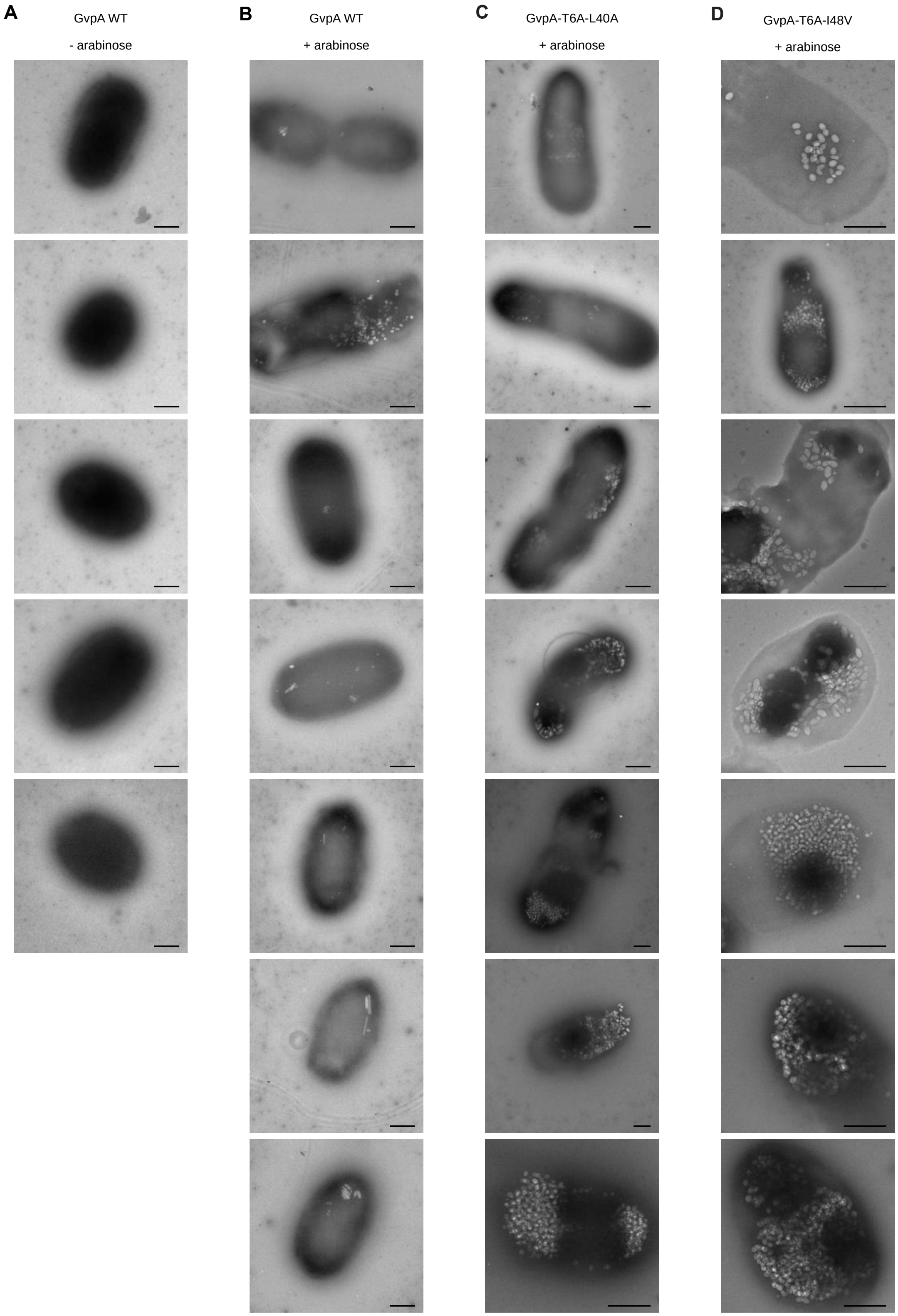

Figure S6. TEM images of *E. coli* cells expressing WT or mutant *A. flos-aquae* GVs. For each sample, 5-25 images were collected; a representative set is shown, ordered from least to most GVs produced per cell. Scale bars 500 nm.

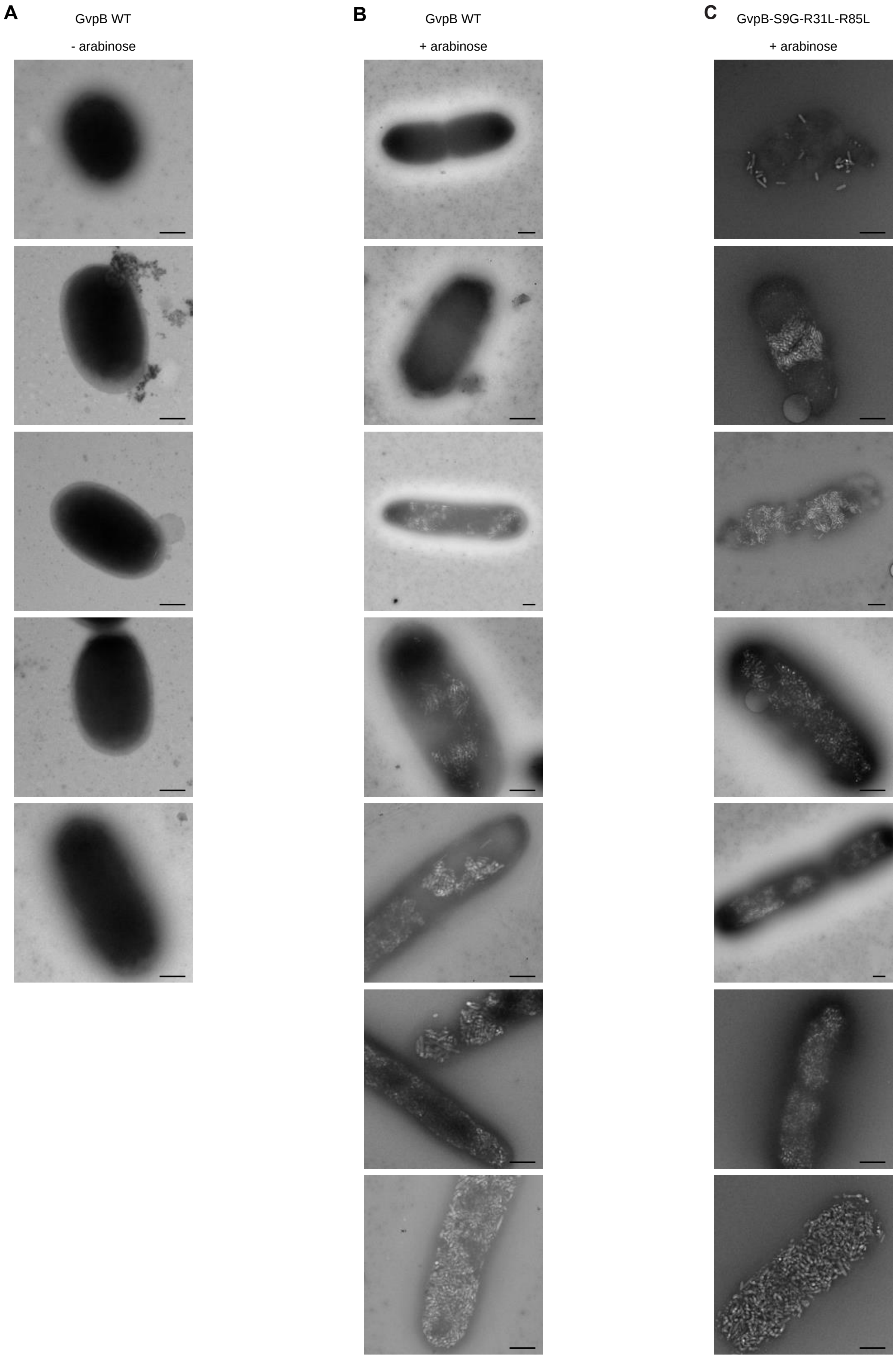

Figure S7. TEM images of *E. coli* cells expressing WT or mutant *B. megaterium* GVs. For each sample, 5-25 images were collected; a representative set is shown, ordered from least to most GVs produced per cell. Scale bars 500 nm.

Supplementary Note 1: Golden Gate reactions
Master mix recipes

Component	Amount per reaction	Stock concentration (NEB)	For 66 reactions
T4 Ligase Buffer	1 uL	10X	66 uL
Hi-T4 DNA Ligase	500 U	400 U/uL	83 uL
BsmBI-v2	15 U	10 U/uL	100 uL
Water	to 5 uL		81 uL

Component	Amount per reaction	Stock concentration (NEB)	For 66 reactions
T4 Ligase Buffer	1 uL	10X	66 uL
Hi-T4 DNA Ligase	500 U	400 U/uL	83 uL
BsaI-HF-v2<br>or<br>BbsI-HF	15 U	20 U/uL	50 uL
Water	to 5 uL		131 uL

To set up reactions, combine 75 ng of the backbone part with 150 ng of each insert part in a PCR tube with 5 uL of the appropriate master mix and fill to 10 uL with water. Miniprepped parts give higher assembly efficiencies than linear PCR products.

Golden Gate thermocycler protocol
20 min 37/42°C
3 minutes 37/42°C
4 minutes 16°C

Cycle 2-3 x30
10 minutes 50°C
10 minutes 80°C
Hold 4°C
